# RASSF1A-Mediated Suppression of Estrogen Receptor Alpha (ERα)-Driven Breast Cancer Cell Growth Depends on the Hippo-Kinases LATS1 and 2

**DOI:** 10.3390/cells10112868

**Published:** 2021-10-24

**Authors:** Sven Roßwag, Jonathan P. Sleeman, Sonja Thaler

**Affiliations:** 1 Department of Microvascular Biology and Pathobiology, European Center for Angioscience (ECAS), Medical Faculty Mannheim, University of Heidelberg, 68167 Mannheim, Germany; Sven.Rosswag@web.de (S.R.); Jonathan.Sleeman@medma.uni-heidelberg.de (J.P.S.); 2 Institute of Biological and Chemical Systems-Biological Information Processing (IBCS-BIP), Karlsruhe Institute of Technology (KIT) Campus Nord, 76344 Eggenstein-Leupoldshafen, Germany

**Keywords:** RASSF1A, ER+ breast cancer, activation of LATS1/2, inhibition of YAP1

## Abstract

Around 70% of breast cancers express the estrogen receptor alpha (ERα). This receptor is of central importance for breast cancer development and estrogen-dependent tumor growth. However, the molecular mechanisms that are responsible for the control of ERα expression and function in the context of breast carcinogenesis are complex and not fully understood. In previous work, we have demonstrated that the tumor suppressor RASSF1A suppresses estrogen-dependent growth of breast cancer cells through a complex network that keeps ERα expression and function under control. We observed that RASSF1A mediates the suppression of ERα expression through modulation of the Hippo effector Yes-associated protein 1 (YAP1) activity. Here we report that RASSF1A-mediated alteration of YAP1 depends on the Hippo-kinases LATS1 and LATS2. Based on these results, we conclude that inactivation of RASSF1A causes changes in the function of the Hippo signaling pathway and altered activation of YAP1, and as a consequence, increased expression and function of ERα. Thus, the inactivation of RASSF1A might constitute a fundamental event that supports the initiation of ERα-dependent breast cancer. Furthermore, our results support the notion that the Hippo pathway is important for the suppression of luminal breast cancers, and that the tumor-suppressor function of RASSF1A depends on LATS1 and LATS2.

## 1. Introduction

The vast majority of breast cancers express the estrogen receptor alpha (ERα). Although this receptor plays a fundamental role in normal breast physiology, abnormal expression and changes in the functional regulation of ERα foster the development and progression of breast cancer [1]. However, the mechanisms that lead to abnormal ERα expression and function remain only partially investigated.

The forkhead box protein 1 (FOXM1) is a transcriptional activator that regulates ERα expression in normal breast tissues, as well as during ERα+ breast cancer initiation, progression and drug resistance. Vice versa, FOXM1 expression is activated by ERα in the presence of estrogens [2]. This is consistent with the observation that elevated expression of FOXM1 in breast cancer strongly correlates with ERα expression [2,3]. FOXM1 is an important regulator of the mitogenic functions of ERα in breast tumor cells, and increased expression of FOXM1 subsequently might contribute to ERα+ breast cancer initiation and progression as FOXM1 triggers cell cycle progression and circumvents induction of senescence [4].

The Ras-associated domain family 1 isoform A (RASSF1A) is frequently inactivated in ERα+ breast carcinomas due to promoter methylation [5,6]. Reconstitution of RASSF1A in ERα+ MCF7 cells led to decreased ERα levels, reduced expression of ERα-target genes with oncogenic functions and reduced sensitivity to estrogen (E2), which is accompanied by the induction of cell cycle arrest and senescence [5]. Furthermore, RASSF1A suppresses FOXM1 expression [7]. These observations suggest that RASSF1A acts as a tumor suppressor in ERα+ breast epithelial cells, in part through the regulation of ERα expression and activity, suppression of ERα-mediated expression of oncogenes as well as through the repression of signaling pathways that are important for E2-independence [5]. However, the molecular mechanisms through which RASSF1A affect ERα expression and function, as well as other proteins that might be important for RASSF1A to mediate its tumor-suppressive functions in breast epithelial cells and during the suppression of breast cancer initiation, remain to be explored.

The Hippo signaling pathway regulates ERα expression and function and suppresses the formation and progression of breast cancer [8,9,10,11,12,13,14]. Comprised of the core kinases MST1/2, LATS1/2 and their target proteins YAP/TAZ [15], this pathway is a master regulator of proliferation, cell death and determination of organ size during development [16,17]. In canonical signaling, activation of the core kinases MST1/2 and LATS1/2 leads to the phosphorylation and inhibition of the downstream effector targets YAP1 and TAZ [15,18,19,20]. When the core kinases are inactive, YAP1 and TAZ are unphosphorylated and translocate into the nucleus to interact with transcription factors such as TEAD1-4, p73, RUNX1/2 or SMADs, NKX2.1, OCT4 and PPARγ [21,22]. The activity of the Hippo-kinases is supported by the WW domain containing the scaffold protein Salvador (SAV1) and the Mps One Binder 1 (MOB1). These stimulate MST1/2 and LATS1/2 phosphorylation, leading to the inhibition of YAP1 and TAZ [18].

In breast tissue, LATS2 can act as an ERα co-repressor [8]. LATS1 and LATS2 restrict the activity of ERα by binding to it and fostering its degradation [9]. Conversely, the ablation of LATS kinases promotes the luminal phenotype and increases the number of bipotent and luminal progenitors, the proposed cells of origin of most human breast cancers [9]. Furthermore, the loss of heterozygosity of LATS1 and frequent copy number loss of LATS2 are often observed in breast cancer [10,11,12,13]. Together, these studies implicate LATS kinases in the regulation of ERα and in the prevention of the initiation and malignant progression of breast cancer.

RASSF1A is an upstream regulator of the Hippo pathway [23,24]. Through its SARAH domain, RASSF1A acts as a scaffold for the MST1/2 kinases and SAV1 [18,25], an interaction that allows RASSF1A to regulate apoptosis, for example, in response to DNA damage or replication stress [26,27]. Mechanistically, the ATM kinase that is activated in response to DNA damage phosphorylates RASSF1A on serine 131 (S131) [28], which promotes the interaction of RASSF1A with MST2, leading to the activation of LATS1-mediated inhibition of CDK2, cell cycle arrest and activation of BRCA2 [27]. Hyperactive RAS also promotes the interaction of RASSF1A with MST1/2, resulting in the activation of MST1/2 kinase activity and the triggering of apoptosis [29,30]. Through its scaffold function, RASSF1A facilitates the phosphorylation of LATS1 by MST2, which leads to YAP1 phosphorylation and its nuclear translocation [29]. The subsequent formation of the YAP1–p73 complex leads to the transcription of pro-apoptotic genes. Thus, RASSF1A inhibits the oncogenic potential of YAP1, for example, through induction of the YAP1 target gene *ANKRD1*, which promotes p53 growth-inhibitory programs via destabilization of MDM2 [31].

Given that RASSF1A is a master regulator of the Hippo pathway and that LATS kinases are implicated in the suppression of breast cancer, we hypothesized that RASSF1A and LATS kinases might cooperate to regulate ERα activity and suppress luminal breast cancer initiation and progression. Consistent with this notion, RASSF1A-mediated modulation of YAP1 was found to be the main mechanism through which RASSF1A suppresses the expression of ERα and FOXM1 [7]. Here we show that RASSF1A depends on LATS1 and LATS2 for the execution of its tumor-suppressor functions in ERα-driven breast cancer cells and suggests that the mutual interaction between RASSF1A and the Hippo-kinases LATS1 and LATS2 is important for the suppression of ERα+ breast cancers. Furthermore, we observed the pharmacological inhibition of YAP1 phenocopies RASSF1A-mediated suppression of ERα and FOXM1 expression, suggesting that drugs that target YAP1 might compensate for the loss of RASSF1A function in ER+ breast cancer cells.

## 2. Results

### 2.1. RASSF1A Decreases YAP1 Protein Levels and Inhibits FOXM1 and ERα Expression

**Figure 1:**
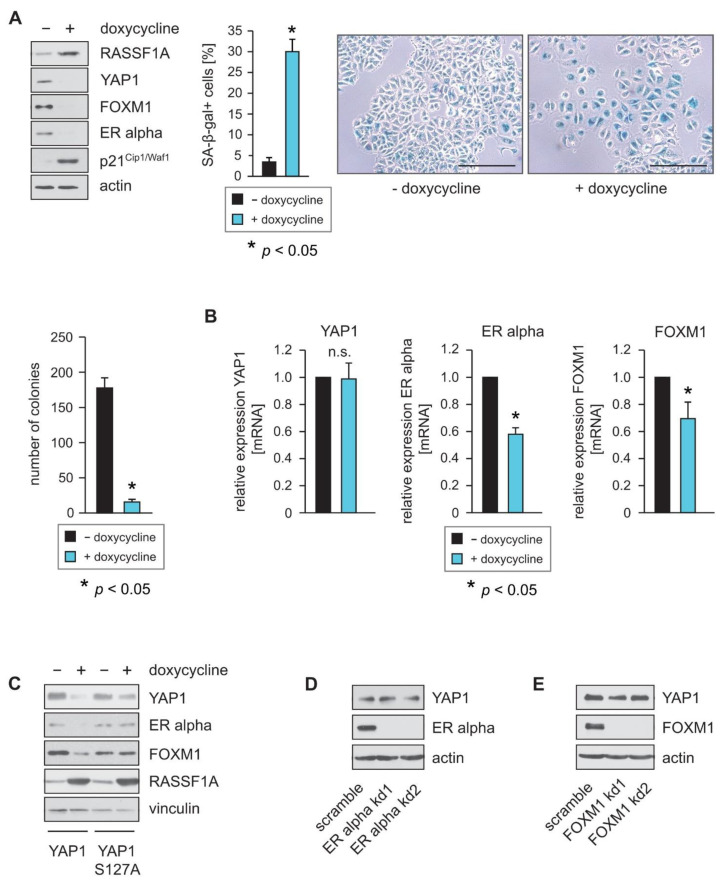
RASSF1A decreases YAP1 protein levels and inhibits FOXM1 and ER alpha expression. (**A**) Expression of RASSF1A in conditional RASSF1A MCF7 cells was induced by culturing cells in the presence of 1 µg/mL doxycycline. Cell extracts from induced and non-induced conditional RASSF1A cells were prepared 48 h after doxycycline administration and were analyzed by Western blotting using the indicated antibodies (left panel). Conditional RASSF1A cells were plated at equal densities and grown for 5 days in the presence or absence of doxycycline as indicated. RASSF1A-induced senescence was subsequently monitored by SA-β-gal staining (right panel). Bars = 100 µm. Quantification of senescent cells was achieved by counting (middle panel). Mean values ± s.d. of six independent experiments are shown. *p*-Values < 0.05 are indicated by asterisks. Equal numbers of conditional RASSF1A cells were plated on six-well plates and grown for 9 days in the presence or absence of doxycycline as indicated. Quantification of colonies was achieved by counting (left lower panel). (**B**) RASSF1A downregulates transcription of ERα and FOXM1 but does not change transcription of YAP1. Conditional MCF7 cells were grown in the presence or absence of 1 µg/mL doxycycline as indicated. mRNA was harvested 48 h after doxycycline administration. ERα, FOXM1 and YAP1 transcript levels were analyzed by quantitative PCR. Mean values ± s.d. of four independent experiments are shown. *p*-values < 0.05 are indicated by asterisks. (**C**) Conditional MCF7 cells grown in the presence or absence of 1 µg/mL doxycycline were transfected with a wild-type YAP1 or a YAP1 S127A expression construct as indicated. Cell extracts were prepared 32 h after transfection. Western blots of lysates were probed with the indicated antibodies. (**D**,**E**) YAP1 expression in MCF7 cells is dependent on ERα and FOXM1. Stable knockdown (kd) of FOXM1 and ERα was performed in conditional MCF7 cells using shRNAs (FOXM1 kd1 and FOXM1 kd2 or ER alpha kd1 and ER alpha kd2). Cell extracts were prepared 48 h after lentiviral transduction. Western blots of lysates from the two independent FOXM1 shRNAs (FOXM1 kd1 and FOXM1 kd2) or ERα shRNAs (ER alpha kd1 and ER alpha kd2) in MCF7 cells were probed with the indicated antibodies (left panels). Neither knockdown of FOXM1 nor knockdown of ERα led to reduced expression of YAP1. mRNA was harvested 48 h after lentiviral transduction. ERα, FOXM1 and YAP1 transcript levels were analyzed by quantitative PCR. Mean values ± s.d. of three independent experiments are shown (right panels).

As RASSF1A is a key regulator of the Hippo pathway, and Hippo signaling plays an important role in the control of ERα function and the suppression of luminal breast cancer, we hypothesized that RASSF1A may exert some of its suppressive effects on ERα and FOXM1 through the Hippo pathway, for example, through the Hippo effector YAP1. Consistent with this hypothesis, the induction of RASSF1A expression in MCF7 and T47D cells that conditionally express RASSF1A upon the addition of doxycycline (RASSF1A-conditional MCF7 or T47D cells) decreased YAP1 levels, reduced the expression of ERα and FOXM1 proteins and suppressed colony formation and the induction of cellular senescence (Figure 1A and Supplementary Figure S1A). Further analysis using qPCR showed that the transcription of ERα and FOXM1 is reduced following RASSF1A induction, whereas the transcription of YAP1 is neither affected in MCF7 (Figure 1B) nor in T47D cells (Supplementary Figure S1B), suggesting that RASSF1A suppresses YAP1 through protein destabilization.

LATS1 and 2 kinases directly phosphorylate YAP1 at S127 [19,20]. Phosphorylated YAP1 binds to 14-3-3 proteins, which retain YAP1 in the cytoplasm through blocking its nuclear import, thereby inhibiting YAP1 function [19]. Retention in the cytoplasm subsequently leads to increased proteasomal destruction of YAP1. The mutation of YAP1 serine 127 to alanine (S127A) renders YAP1 resistant to LATS1 and 2 induced phosphorylation, resulting in increased nuclear import of YAP1 and elevated YAP1 activity. To test the hypothesis that RASSF1A suppresses YAP1 through protein destabilization, we next overexpressed wild-type YAP1 or YAP1 S127A in RASSF1A-conditional MCF7 cells. Consistent with our hypothesis, induction of RASSF1A led to a stronger reduction in ectopically expressed wild-type YAP1 protein levels compared to YAP1 S127A levels (Figure 1C). Furthermore, we observed a stronger relative reduction in ERα and FOXM1 protein levels upon RASSF1A expression in wild-type YAP1-expressing conditional MCF7 cells than in the YAP1 S127A conditional MCF7 cells (Figure 1C). These data are consistent with the notion that RASSF1A suppresses YAP1 through protein stabilization and activation of the Hippo pathway. They are also consistent with published data that show that a major consequence of RASSF1A depletion is a nuclear accumulation of YAP1 [32,33,34] and that RASSF1A increases degradation and thereby suppresses YAP1, whereas loss of RASSF1A leads to increased activity and increased expression of YAP1-target genes.

As ERα and FOXM1 are both transcriptional activators, we next investigated whether reduced expression of YAP1 might additionally be a consequence of RASSF1A-mediated suppression of either ERα or FOXM1. To determine whether loss of ERα or FOXM1 expression causes reduced expression of YAP1, we used stable knockdown of ERα and FOXM1 in parental MCF7 cells. Reduced expression of ERα and FOXM1 was verified by Western blotting (Figure 1D,E). In contrast to the induction of RASSF1A expression, neither knockdown of ERα nor knockdown of FOXM1 caused reduced YAP1 protein levels. Taken together, these observations are consistent with the notion that suppression of the Hippo effector YAP1 by RASSF1A plays an important role in RASSF1A-induced cell cycle arrest and senescence, as well as in RASSF1A-mediated suppression of ERα-driven breast cancer.

### 2.2. YAP1 Knockdown Phenocopies the Effects of RASSF1A

**Figure 2:**
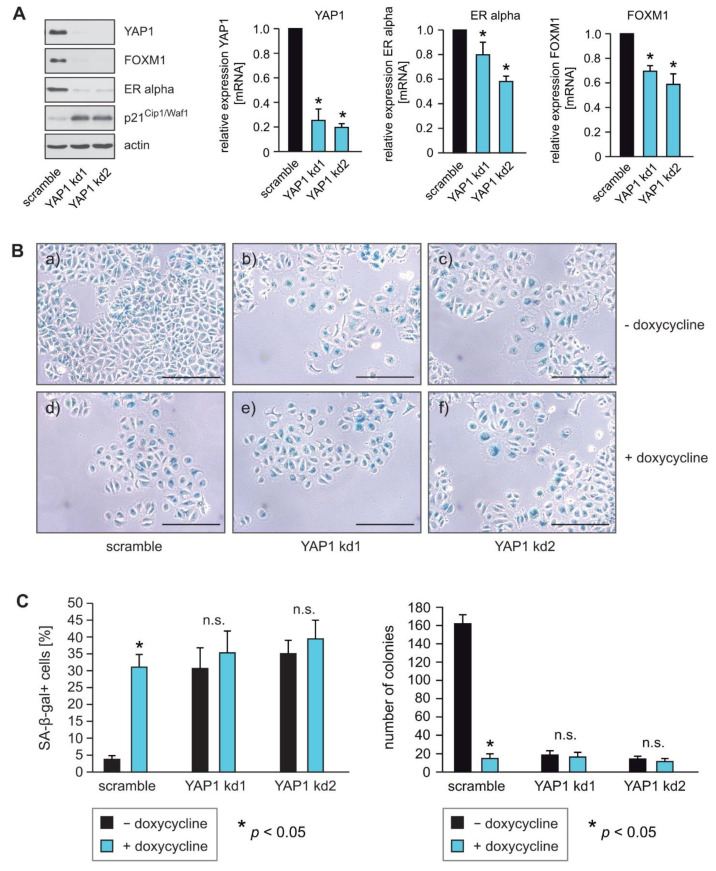
YAP1 knockdown phenocopies the effects of RASSF1A. (**A**) Stable knockdown of YAP1 was performed in conditional MCF7 cells using shRNAs (YAP1 kd1 and YAP1 kd2). Cell extracts were prepared 48 h after lentiviral transduction. Western blots of lysates from the two independent YAP1 shRNAs (YAP1 kd1 and YAP1 kd2) cells were probed with the indicated antibodies (left panel). Knockdown of YAP1 in MCF7 cells using the shRNAs YAP1 kd1 and YAP1 kd2 was performed and mRNA was prepared 48 h after lentiviral transduction. Transcript levels of the indicated genes were analyzed by quantitative PCR. Mean values ± s.d. of three independent experiments are presented. *p*-Value < 0.05 is indicated by asterisks. (**B**) Equal numbers of conditional RASSF1A cells were plated on six-well plates and transduced with equal amounts of lentiviral particles carrying non-targeted shRNA (scramble) (a+b), shRNA YAP1 kd1 (b+d) or shRNA YAP1 kd2 (c+f) lentiviral particles. 48 h after transduction conditional RASSF1A scramble, YAP1 kd1 and YAP1 kd2 cells were cultured for either 5 or 9 days in the presence or absence of 1 μg/mL doxycycline as indicated. For quantification of SA-β-gal positive cells, cells were fixed and stained after 5 days. For quantification of colonies, cells were fixed after 9 days. RASSF1A-induced senescence was subsequently monitored by SA-β-gal staining (upper panel). Bars = 100 μm. (**C**) Quantification of senescent cells was achieved by counting (left panel). Mean values ± s.d. of three independent experiments are shown. *p*-Values < 0.05 are indicated by asterisks. Quantification of colonies was achieved by counting (right panel). Mean values ± s.d. of three independent experiments are presented. *p*-Values < 0.05 are indicated by asterisks.

To determine whether loss of YAP1 expression functionally phenocopies RASSF1A expression, we used stable knockdown of YAP1 in RASSF1A-conditional MCF7 cells. Reduced expression of YAP1 was verified by Western blotting and qPCR (Figure 2A). Similar to the induction of RASSF1A expression, knockdown of YAP1 caused reduced expression of ERα and FOXM1 and the induction of senescence (Figure 2A,B). No significant differences in the induction of senescence could be observed between RASSF1A-expressing and non-RASSF1A-expressing YAP1 knockdown cells (Figure 2B,C, left panel). Taken together, these observations are consistent with the notion that the suppression of YAP1 expression by RASSF1A plays a pivotal role in RASSF1A-induced cell cycle arrest and senescence, and mechanistically explains how the loss of RASSF1A contributes to ERα+ breast cancer initiation and progression.

### 2.3. Pharmacological Inhibition of YAP1 by Dasatinib Causes Inhibition of ERα and FOXM1 Expression Similar to Knockdown of YAP1

**Figure 3:**
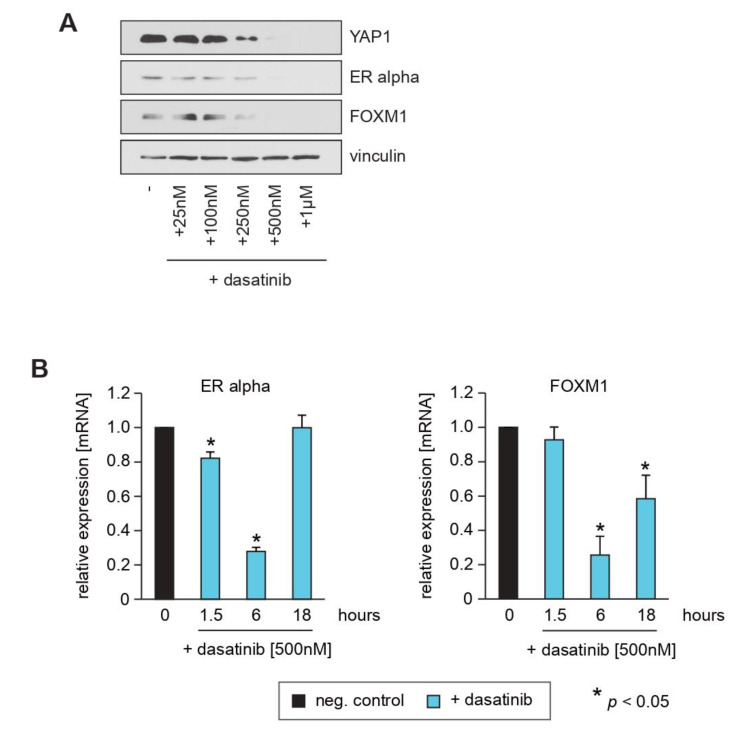
Pharmacological inhibition of YAP1 by dasatinib causes inhibition of ERα and FOXM1 expression similar to knockdown of YAP1. (**A**) Conditional RASSF1A MCF7 cells were cultured in the absence or presence of the indicated concentrations of dasatinib. After 16 h, cells were harvested and subsequent lysates were analyzed by immunoblotting using the indicated antibodies. (**B**) Dasatinib downregulates transcription of ERα and FOXM1 and causes decreased amounts of YAP1 protein. Mean values ± s.d. of three independent experiments are presented. *p*-Values < 0.05 are indicated by asterisks.

The SRC tyrosine kinase activates YAP1 and thereby drives tumor onset, growth, progression and metastasis. As the SRC family inhibitor dasatinib is an effective YAP1 inhibitor [35,36,37], we next investigated whether pharmacological inhibition of YAP1 has a similar effect on ERα and FOXM1 expression as YAP1 knockdown. Similar to the knockdown of YAP1 through shRNAs, dasatinib caused reduced expression of ERα and FOXM1 in a dose and time-dependent manner, as evidenced by Western blotting (Figure 3A) and qPCR (Figure 3B). Note that the transcription of these genes rebounds after 18 h due to the short half-life of dasatinib in an aqueous solution. Furthermore, we observed that treatment of MCF7 and T47D cells with dasatinib causes an increase in cells within the G1-phase and a decrease in cells in G2/M (Supplementary Figure S2A–D), suggesting the induction of cell cycle arrest. Taken together, these results are consistent with the notion that pharmacological suppression of YAP1 phenocopies the effect of RASSF1A expression in ERα expressing breast cancer cells.

### 2.4. LATS1 and LATS2 Knockdown Circumvent RASSF1A-Mediated Suppression of YAP1, ERα and FOXM1 Expression

**Figure 4:**
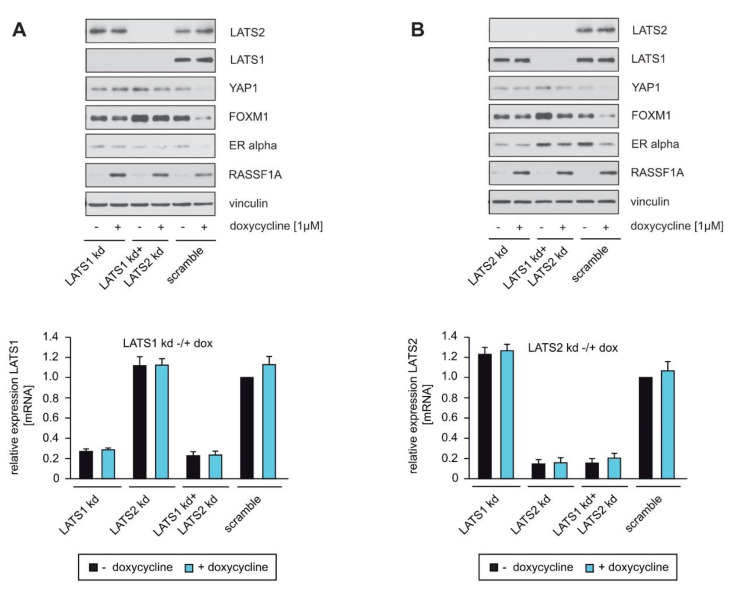
LATS1 and LATS2 knockdown circumvent RASSF1A-mediated suppression of YAP1, ERα and FOXM1 expression. (**A**,**B**) Stable knockdown of LATS1, LATS2 and LATS1+LATS2 was performed in MCF7 conditionally expressing RASSF1A cells using shRNAs (LATS1 kd and LATS2 kd). For Western blotting, SA-β-gal staining, colony-forming assays and qPCR analysis, equal numbers of conditional MCF7 cells were plated on either 10 cm cell culture or six-well plates and transduced with equal amounts of lentiviral particles carrying non-targeted shRNA (scramble), shRNA LATS1 kd or shRNA LATS2 kd lentiviral particles. Then, 48 h after transduction, conditional RASSF1A MCF7 cells were cultured in the absence or presence of 1 μg/mL doxycycline. Cell lysates and mRNA were prepared 48 h after doxycycline treatment. Western blots of lysates from the conditional RASSF1A MCF7 scramble, LATS1 kd, LATS2 kd and LATS1+LATS2 double knockdown (LATS1 kd+LATS2 kd) cells were probed with the indicated antibodies (left panels). Efficiency of LATS1, LATS2 and LATS1+LATS2 double knockdowns were also quantified by qPCR. Mean values ± s.d. of two independent experiments are presented.

YAP1 is a direct target of the Hippo core kinases LATS1 and LATS2 [15,18,19,20]. Based on our finding that RASSF1A decreases the amount of wild-type YAP1 but not of mutant YAP1 S127A (Figure 1C), we hypothesized that RASSF1A-mediated suppression of YAP1 depends on the Hippo-kinases LATS1 and/or LATS2. To investigate whether LATS1 and/or LATS2 are indeed responsible for RASSF1A-mediated suppression of YAP1, ERα and FOXM1 expression in ERα+ breast cancer cells, we employed stable knockdown of either LATS1, LATS2 or LATS1+LATS2 in RASSF1A-conditional MCF7 cells. The efficacy of shRNAs against LATS1 and LATS2 in reducing LATS1 and LATS2 expression was determined by Western blotting or qPCR. shRNAs that generated a strong reduction in LATS1 and LATS2 expression were used to perform functional analyses. RASSF1A-conditional cells with knockdown of either LATS1, LATS2 or LATS1+LATS2 exhibited little or even no reduction of YAP1, ERα and FOXM1 in the presence of RASSF1A in comparison to their negative-control scrambled counterparts (Figure 4A). qPCR revealed that the reduction of LATS1 and LATS2 expression was comparable in each condition (Figure 4B). These results are consistent with the notion that the Hippo-kinases LATS1 and LATS2 are of central importance for RASSF1A-mediated suppression of YAP1, ERα and FOXM1 expression.

### 2.5. Knockdown of LATS1 and LATS2 Circumvents RASSF1A-Mediated Induction of Senescence

**Figure 5:**
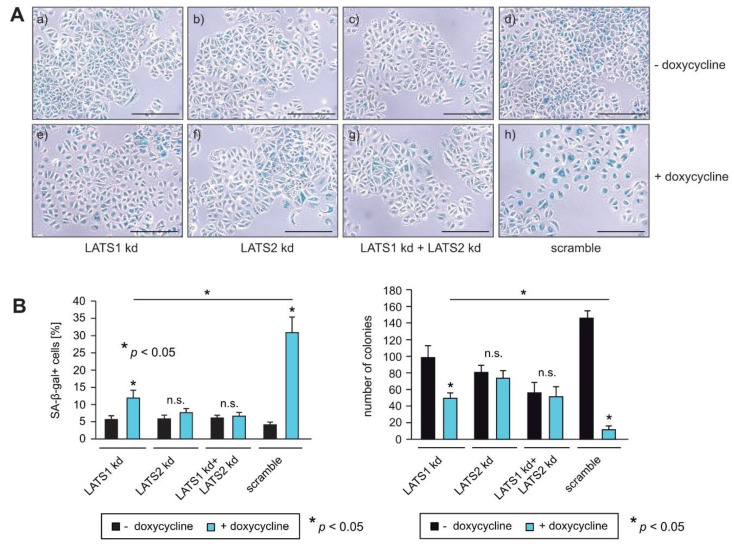
Knockdown of LATS1 and LATS2 circumvent RASSF1A-mediated induction of senescence. (**A**,**B**) RASSF1A-induced senescence in conditional RASSF1A MCF7 scramble, LATS1 kd, LATS2 kd and LATS1+LATS2 double knockdown cells was monitored by SA-β-gal staining (right panel). Cells were cultured in the absence (a–d) or presence of 1 µg/mL doxycycline (e–h) as indicated. Bars = 100 µm. (**B**) Quantification of senescent cells was achieved by counting (left panel). Mean values ± s.d. of three independent experiments are shown. *p*-Values < 0.05 are indicated by asterisks. Quantification of colonies was achieved by counting (left panel). Mean values ± s.d. of four independent experiments are presented. *p*-Values < 0.05 are indicated by asterisks.

**Figure 6:**
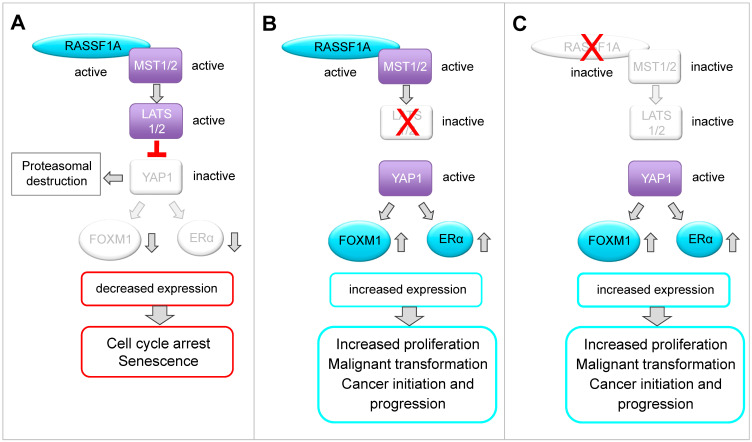
Schematic model of a possible molecular network between RASSF1A, the Hippo-kinases LATS1 and LATS2, the Hippo kinase effector YAP1, FOXM1 and ERα based on the findings in this paper and on the published literature. (**A**) RASSF1A is a key regulator of the Hippo signaling pathway. In this study, we observed that RASSF1A-mediated suppression of YAP1 and YAP1 inhibition correlates with reduced expression of FOXM1 and ERα in ERα-positive breast cancer cells. Furthermore, we demonstrate that decreased expression of YAP1, FOXM1 and ERα correlates with induction of senescence, reduced colony formation and inhibition of cancer cell growth. FOXM1 and ERα play important roles in the regulation of proliferation, function and development of normal breast epithelium. Increased expression and aberrations in the function or expression of these proteins are associated with luminal breast cancer initiation, progression and drug resistance. Based on our findings, we suggest that in normal breast tissue, RASSF1A keeps expression and function of FOXM1 and ERα under control, thereby suppressing luminal breast cancer initiation and progression in a LATS1-, LATS2- and YAP1-dependent manner. In normal breast epithelium, RASSF1A is expressed without inducing senescence. We speculate that RASSF1A only causes down-regulation of FOXM1 and ERα after being activated e.g., through mitogenic stimuli or under the influence of hormones such as estrogen. It was shown that RASSF1A is phosphorylated by ATM kinase as a consequence of DNA damage or mitotic replication stress. Phosphorylation facilitates interaction of RASSF1A with MST2 leading to activation of the Hippo pathway. The outcome of RASSF1A on YAP1 function or stabilization might be dependent on the cellular context. It was reported that RASSF1A causes YAP1-dependent expression of pro-apoptotic genes as a consequence of RASSF1A-mediated activation of the Hippo pathway. However, it was also reported that phosphorylation of YAP1 by LATS1/2 causes its cytoplasmic retention followed by proteasomal destruction, suggesting that RASSF1A might also suppress YAP1 through fostering its proteasomal degradation. Here, we show that RASSF1A decreases the levels of YAP1 and, as a consequence, suppression of FOXM1 and ERα expression and senescence in ERα-driven breast cancer cells. Interestingly, it was reported that YAP1 suppresses RASSF1A by fostering its proteasomal destruction. Thus, it is conceivable that RASSF1A and YAP1 mutually antagonize each other and that a regulatory feedback loop exists between both proteins. Loss of RASSF1A or aberrations in the function of the Hippo-kinases LATS1 and 2 might shift the balance towards an increased activation of YAP1, FOXM1 and ERα fostering luminal breast cancer initiation and progression. (**B**) We observed that RASSF1A-mediated suppression of YAP1, FOXM1 and ERα depends on the Hippo-kinases LATS1 and LATS2. This observation is in accordance with reports showing that LATS kinases are important for control of ERα activity and in suppression of luminal breast cancers. (**C**) We observed in the absence of RASSF1A that the levels of the Hippo pathway effector YAP1 are increased and that YAP1 affects expression and activity of ERα and FOXM1 even in the presence of LATS1 and LATS2. Based on our findings, we suggest that RASSF1A and LATS1 and 2 cooperate in suppressing YAP1 and inhibiting FOXM1 and ERα expression. FOXM1 is a key regulator of cell cycle progression and an activator of ERα expression. Thus, deregulated FOXM1 expression leads to uncontrolled proliferation and resistance against senescence.

To confirm that LATS1 and LATS2 are important for RASSF1A-mediated growth arrest, equal numbers of RASSF1A-conditional LATS1, LATS2 and LATS1+LATS2 knockdown and scrambled control cells were cultured in the presence or absence of doxycycline. Approximately 5 days later, cells were fixed and stained for the detection of senescence. In parallel, equal numbers of cells were seeded on six-well plates and 12 days later colonies were fixed and counted at equivalent time points. Knockdown cells displayed less induction of senescence and formed more colonies than negative control cells in the presence of RASSF1A (Figure 5A,B), indicating that LATS1 and LATS2 are required for RASSF1A-mediated growth arrest and senescence. Taken together, these observations are consistent with the notion that LATS1 and LATS2 play a pivotal role in RASSF1A-induced cell cycle arrest and senescence and mechanistically explain how the loss of either RASSF1A and/or inactivation of LATS1 and LATS2 contributes to ERα+ breast cancer initiation and progression (Figure 6).

## 3. Discussion

Over recent years, it has become clear that the negative impact of RASSF1A on breast cancer cell growth is mediated through a complex molecular network. RASSF1A expression suppresses ERα levels, resulting in reduced expression of ERα-target genes that have oncogenic functions, impaired sensitivity to estrogen, and the induction of cell cycle arrest and senescence [5]. In addition, RASSF1A suppresses FOXM1 expression [7], a transcriptional activator of the ERα gene that is associated with resistance towards endocrine therapies [2,38]. The findings we present here extend the molecular network through which RASSF1A exerts its suppressive effects on breast cancer initiation and progression. Specifically, we show that RASSF1A reduces ERα and FOXM1 expression through a hierarchical pathway in which RASSF1A-mediated activation of the Hippo pathway core kinases LATS1 and LATS2 initially suppresses the Hippo effector YAP1, which subsequently leads to the inhibition of FOXM1 and ERα expression (Figure 6).

Our data implicate the LATS kinases as central mediators of the tumor-suppressive activity of RASSF1A. Consistently, LATS kinases have been implicated in the regulation of ERα activity and in the control of ERα stability [8,9]. In breast tissue, the silencing of LATS2 led to the increased transcriptional activity of ERα, suggesting that LATS2 might act as a transcriptional repressor of ERα that suppresses the expression of ERα target genes [8]. Furthermore, LATS1 and LATS2 interact directly with ERα. In the presence of LATS kinases, ERα is targeted for ubiquitination and proteasomal degradation, while in the absence of LATS-kinases, ERα and the Hippo-effectors YAP1 and TAZ are stabilized [9]. In these studies, the effects of LATS kinases on ERα expression and activity were independent of YAP1/TAZ [8,9]. By contrast, we found that RASSF1A suppresses ERα and FOXM1 expression through a mechanism that is dependent on both LATS and YAP1 (Figure 2A and Figure 4A), suggesting that LATS kinases regulate ERα expression through both YAP1-dependent and -independent mechanisms.

The knockdown of YAP1 reduced colony formation and increased the number of senescent cells in comparison to scramble control counterparts (Figure 2B,C). These data suggest that YAP1 is important for the survival and proliferation of ERα-expressing breast cancer cells, and that aberrations in the Hippo pathway that lead to increased YAP1 activity are likely to foster breast cancer initiation and progression. Furthermore, the knockdown of YAP1 led to increased expression of the cell cycle inhibitor p21^Cip1/Waf1^ (Figure 2A). These findings are in accordance with recently reported findings that pharmacological inhibition of YAP1 or YAP1 depletion led to increased expression of p21 and the induction of senescence [39].

YAP1 can also be activated without inhibition of the Hippo-kinases MST1/2 and LATS1/2 [22], and RASSF1A can regulate YAP1 activity without the full Hippo pathway downstream of TGFβ by restricting the interaction of YAP1 with SMAD2 [40]. Here, we found that knockdown of LATS1, LATS2 or LATS1+LATS2 almost completely circumvented RASSF1A-mediated suppression of YAP1, ERα and FOXM1 expression (Figure 4A) as well as RASSF1A-induced cell growth and senescence (Figure 5A,B), indicating that LATS kinases are of particular importance for the tumor suppressor function of RASSF1A in ERα-driven breast cancer cells. Our results are therefore consistent with the notion that RASSF1A suppresses YAP1 through activation of the Hippo pathway, as RASSF1A activates the Hippo core kinases MST1/2 and LATS1/2. Furthermore, these data suggest that reduced activity of Hippo-kinases such as LATS1 and LATS2 could serve to circumvent RASSF1A-mediated tumor suppressive mechanisms.

Knockdown of LATS1 and LATS2 by itself partially decreased colony formation, and the most potent shRNA against LATS2 also induced cellular senescence (Figure 5B and Supplementary Figure S3). Consistently, a strong reduction in LATS1 has also been reported to induce senescence by others [41]. These observations suggest that the degree of loss of LATS1 and LATS2 activity may be decisive for the outcome of reduced LATS expression and activity. Thus, mechanisms that lead to only a partial loss or altered functionality of the LATS proteins might be required to abrogate the RASSF1A-mediated suppression of YAP1, ERα and FOXM1, thereby fostering the initiation and progression of luminal breast cancer. Consistent with this notion, the loss of heterozygosity of LATS1 [10,11,12] and frequent copy number loss of LATS2 [13] has been reported in breast cancer, suggesting that partial but not complete inactivation of LATS kinases is important for breast cancer development. In this context, it is notable that a recent study reported that double knockout of LATS1 and 2 in MCF7 cells using CRISPR-Cas9 led to decreased *ESR1* mRNA and ERα protein via YAP and TAZ, and decreased cell growth in vitro and in vivo [42]. By contrast, others reported that knockdown of LATS1 and 2 by shRNAs upregulated ERα protein, and that the expression of full-length LATS1 or LATS1 lacking the kinase domain decreased ERα independently of YAP1 and TAZ [7]. Although partially contradictory, these studies nevertheless suggest that partial but not complete inactivation of LATS kinases is important for breast cancer development, and that LATS1 and 2 do not only act as tumor suppressors [43], consistent with other observations [10,11,12,13].

Here, we show that RASSF1A decreases the levels of YAP1, and as a consequence, the suppression of FOXM1 and ERα expression and senescence in ERα-driven breast cancer cells. We also demonstrate that knockdown of YAP1 decreased the expression of FOXM1 and ERα, phenocopying the effects of RASSF1A (Figure 2A–C) [9]. Since we used the cells shortly after lentiviral transduction, we can exclude clonal effects. As YAP1 is frequently upregulated in human cancers, YAP1 is often considered to be an oncogene rather than a tumor suppressor gene. Nevertheless, YAP1 also has tumor-suppressive functions. The hippo core kinases are central modulators of p53 and YAP1 activity. Although YAP1 can facilitate both pro-and anti-tumorigenic activities, it is suggested that LATS kinases are major regulators that maintain wild-type p53 activity and balance the tumor-promoting functions of YAP1 through cooperating with RASSF1A [44,45]. Thus, RASSF1A can use YAP1 to activate tumor suppressor genes, induce apoptosis and inhibit the oncogenic potential of YAP1 [29,31,34]. It is therefore conceivable that the tumor-promoting or cancer suppressive functions of YAP1 might be dependent on the presence of RASSF1A, and that RASSF1A modulates the function of YAP1 such that it acts as a tumor suppressor. On one hand, RASSF1A causes YAP1-dependent expression of pro-apoptotic genes as a consequence of RASSF1A-mediated activation of the Hippo pathway [29]. On the other hand, phosphorylation of YAP1 by LATS1/2 causes cytoplasmic retention and proteasomal destruction of YAP1 [15,18,19,20], suggesting that RASSF1A can suppress YAP1 through cytoplasmic retention and subsequently foster its proteasomal degradation. This notion is supported by our observation that RASSF1A decreases wild-type YAP1 but not mutant YAP1 S127A, which is resistant to LATS1- and 2-induced phosphorylation, resulting in increased nuclear import and elevated YAP1 activity (Figure 1C). Notably, inhibition of YAP1 by dasatinib in the context of rhabdomyosarcoma is only successful in combination with DNA methyltransferase inhibitors (DNMTi) that upregulate RASSF1 and RASSF5 by promoter demethylation, resulting in the activation of canonical Hippo signaling and the inactivation of YAP1 by phosphorylation [46]. Remarkably, the effects of DNMTi-mediated RASSF1 activation were rescued by the expression of constitutively active YAP (S127A) [46], suggesting that RASSF1A-mediated inhibition of YAP1 is Hippo signaling dependent.

Interestingly, YAP1 suppresses RASSF1A by fostering its proteasomal destruction [40]. Thus, it is conceivable that RASSF1A and YAP1 mutually antagonize each other and that a regulatory feedback loop exists between both proteins. The loss of RASSF1A or aberrations in the function of the Hippo-kinases LATS1 and 2 might shift the balance towards increased activation of YAP1, FOXM1 and ERα, fostering luminal breast cancer initiation and progression.

RASSF1A is expressed in normal breast epithelium without inducing senescence. We speculate that RASSF1A only causes down-regulation of FOXM1 and ERα after being activated, for example, through phosphorylation by the ATM kinase as a consequence of DNA damage or mitotic replication stress [27,28]. Phosphorylation of RASSF1A facilitates its interaction with MST2, leading to activation of the Hippo pathway and subsequently to senescence or apoptosis.

The results we present here are consistent with findings made in certain soft tissue sarcomas. In this context, FOXM1 expression was found to be increased by YAP1, and YAP1-dependent expression of FOXM1 was necessary for tumorigenesis [47]. Furthermore, FOXM1 was shown to directly interact with the YAP1 transcriptional complex via TEAD1, resulting in the co-regulation of numerous critical proliferation targets that enhance sarcoma progression [47]. These findings demonstrate that YAP1 acts as an oncogene through increasing the expression of FOXM1, and that both proteins together act as oncogenes through increasing the expression of further genes that are important to drive tumorigenesis.

The inactivation of RASSF1A as well as alterations in the Hippo pathway occur frequently in breast cancer. RASSF1A and the Hippo-kinases LATS1/2 are of particular importance for the suppression of ERα-driven breast cancer [5,6,7,8,9]. On one hand, the loss of RASSF1A causes aberrant Hippo pathway activity [27,29,30]. On the other hand, the loss or inactivation of Hippo pathway components circumvents the RASSF1A tumor-suppressor function [29]. Drugs that target the oncogenic function of the Hippo pathway downstream effectors YAP1 and TAZ are therefore possible tools to compensate for the loss of Hippo-kinases, and to phenocopy RASSF1A function within cancer cells with either the loss of RASSF1A or defects in the Hippo pathway. It is therefore significant that we observed that pharmacological inhibition of YAP1 via the use of the SRC tyrosine kinase inhibitor dasatinib phenocopied RASSF1A-mediated suppression of ERα and FOXM1 expression. Our data therefore provide support for the development of drugs that target YAP1 as a therapeutic opportunity for the treatment of ERα-driven breast cancer. However, as recently reported for rhabdomyosarcoma [46], it is possible that dasatinib might not be sufficient to phenocopy the effects of RASSF1A, and that the combination of dasatinib with RASSF1A-activating drugs such as DNMTi is needed to suppress YAP1 activity and YAP1-mediated FOXM1 and ERα expression. Notably, it was reported that sequential targeting of YAP1 and p21 led to the elimination of senescent tumor cells [39]. As senescent cells may contribute to disease recurrence after cancer therapy, the potential clinical application of YAP1 inhibition together with RASSF1A-activating drugs may also require sequential targeting of p21 to shift senescent cells into apoptosis.

## 4. Materials and Methods

### 4.1. Plasmids and Reagents

Details about the antibodies, shRNA, primer sequences and reagents used in this study can be found in Supplementary Table S1. The plasmids p2xFLAGhYAP1-S127A and p2xFLAGhYAP1 were gifts from Marius Sudol (Addgene plasmid # 17790; http://n2t.net/addgene:17790, accessed on 22 June 2020; RRID: Addgene_17790) and (Addgene plasmid # 17791; http://n2t.net/addgene:17791, accessed on 22 June 2020; RRID: Addgene_17791) [48].

### 4.2. Cell Lines, Cell Culture and Transient Transfection

The human breast cancer cell lines MCF7 and T47D and the human embryonic kidney cell line HEK293T were purchased from the American Type Culture Collection (ATCC, Manassas, VA, USA). Unless otherwise indicated, MCF7 and T47D cells were maintained in RPMI. HEK293T cells were maintained in DMEM. RPMI and DMEM were supplemented with 10% fetal bovine serum (Takara Clontech, Heidelberg, Germany), 1% L-glutamine and 1% penicillin/streptomycin. For the production of RASSF1A-conditional MCF7 and T47D cells, the Tet-regulated transactivator rtTA-M2 was cloned into the retroviral expression vector pQCXIP (Takara Clontech) and human RASSF1A was cloned into the retroviral vector pRevTRE (Takara Clontech). After transduction, RASSF1A-conditional cell lines were achieved through culturing transduced MCF7 and T47D cells in the presence of puromycin and hygromycin B (MCF7 cells: 0.125 µg/mL puromycin and 125 µg/mL hygromycin B; T47D cells: 0.05 µg/mL puromycin and 15 µg/mL hygromycin B). The induction of RASSF1A expression was achieved via the addition of doxycycline (1 µg/mL). For all experiments, pooled, transduced, selected conditional MCF7 and conditional T47D cells were used. The selection of conditional RASSF1A cells and all experiments were performed in RPMI supplemented with Tet-system-approved FBS (ThermoFisher, Waltham, MA, USA), 1% L-glutamine and 1% penicillin/streptomycin. Puromycin and doxycycline were purchased from Sigma-Aldrich (Taufkirchen, Germany) and hygromycin B from Merckmillipore (Darmstadt, Germany). For all experiments, cells were maintained at 37 °C in a humidified 5% CO_2_ incubator. Transient transfection of the MCF7 cells, with the expression constructs p2xFLAGhYAP1 and p2xFLAGhYAP1-S127A, was performed on 10 cm cell culture dishes with Fugene 6 (Promega, Heidelberg, Germany) according to the manufacturer’s instructions.

### 4.3. Lentiviral Production and Viral Transduction

For the generation of lentiviruses, 1.8 × 10^6^ cells of the packaging cell line HEK293T were seeded on 10 cm cell culture dishes previously coated with 10 µg/mL human plasma fibronectin (Merckmillipore). After 12–14 h, cells were transfected using calcium phosphate transfection. To this end, 1.5 µg pVSV-G, 1.5 µg pRSV Rev, 1.5 µg pMDLg/pRRE and 10 µg of the respective TRC pLKO shRNA or scramble plasmids were adjusted with distilled water to a final volume of 200 µL in a sterile FACS tube. Afterwards, 50 µL of 2M CaCl_2_ were added to the diluted plasmids, then 250 µL 2 × HBS buffer were pipetted dropwise while vortexing the mixture. After 20–30 min, the plasmid calcium phosphate complexes were pipetted drop by drop onto the packaging cells. After 14–18 h, lentiviruses were harvested by collecting the medium conditioned by the packaging cells. Afterwards, the conditioned medium was filtered through a CME 0.45 µm syringe filter (Roth GmbH, Karlsruhe, Germany). Target cells were transduced with the lentiviruses for 18–24 h in the presence of hexamethrine bromide (5–8 µg/mL). For all experiments, pooled transduced cell clones were used.

### 4.4. Western Blotting

Cells for Western blotting were lysed with RIPA buffer (50 mM Tris-HCl pH 8.0, 150 mM NaCl, 1% NP-40, 0.5% sodium deoxycholate, 0.1% SDS). Lysis buffer was supplemented with a 1× protease inhibitor cocktail (Complete, Roche) and phosphatase inhibitor cocktail (Sigma-Aldrich). The protein concentration was determined using the Bio-Rad Protein Assay Dye Reagent (Bio Rad, Philadelphia, PA, USA). Cell lysates (25–40 µg) were subjected to SDS-PAGE, transferred to nitrocellulose membranes (Bio Rad) and blocked for 1 h at room temperature with 5% milk in PBS-0.05% Tween 20. After blocking, membranes were incubated overnight at 4 °C with primary antibodies followed by 3× washing with 5% milk in PBS-0.05% Tween 20. Membranes were than incubated with secondary HRP-coupled anti-rabbit (1:2000) or anti-mouse (1:1000) antibodies for 1–2 h at room temperature. All antibodies are listed in Supplementary Materials. The chemiluminescence detection reagents Pierce^TM^ ECL Western blotting substrate (ThermoFisher) or SuperSignal^TM^ West Pico PLUS chemoluminiscent (ThermoFisher) were used to visualize protein bands on X-ray films.

### 4.5. SA-β-Gal Staining

Cells were seeded on six-well plates, washed with PBS and fixed for 5–15 min in 2% formaldehyde/0.2% glutaraldehyde. After fixation, cells were washed three times with PBS and incubated at 37 °C (without CO_2_) in freshly prepared senescence-associated β-Gal (SA-β-Gal) staining solution [1 mg of 5-bromo-4-chloro-3-indolyl β-D-galactoside (X-Gal)] per mL (stock = 20 mg of dimethylformamide per mL)/40 mM citric acid/sodium phosphate, pH 6.0/5 mM potassium ferrocyanide/5 mM potassium ferricyanide/150 mM NaCl/2 mM MgCl_2_. Cells were incubated in the staining solution for 14–16 h [49]. The number of SA-β-gal+ cells was quantified from at least five random fields per sample, with each field containing a minimum of 200 cells. SA-β-gal+ cells were quantified by a single evaluator in a blinded manner. The percentage of SAβ-gal+ cells calculated using the formula (SA-β-gal+ cells/total cells in a field) × 100%.

### 4.6. Colony Formation Assay

Cells were seeded in six-well plates as triplicates or quadruplicates at a density of 5 × 10^3^–1 × 10^4^ cells per well. After 9–10 days, cells were fixed and stained with Coomassie dye or with 0.5% crystal violet for 20 min at room temperature. All colonies on each plate were quantified by counting single colonies by a single evaluator in a blinded manner.

### 4.7. Quantitative RT-PCR Analysis and Primer Design

Total RNA was prepared using the TRIZOL reagent (Life Technologies, Carlsbad, CA, USA) according to the manufacturer’s protocol. The concentration and purity of the RNA were determined using a Nanodrop^TM^ spectrometer (ThermoFisher). cDNA was synthesized from total RNA (1 µg) using Revert Aid H Minus Reverse Transcriptase (Life Technologies) with random hexamer primers (Life Technologies). Quantitative real-time PCR was performed in a reaction volume of 20 µL containing 1 × GoTaq qPCR Master Mix (Promega) and 25 ng cDNA, using a Mx3005P Real-Time PCR System with MxPro qPCR software (Agilent). The threshold cycle (Ct) value for each gene was normalized to the Ct value for RibPO. The relative mRNA expression was calculated using the 2^ΔΔCt^ method. Primers were designed using Ensembl and PrimerQuest. Sequences of all primers used are listed in under 4.10. and in Supplementary Table S1.

### 4.8. PI Staining and FACS Analysis

MCF7 and T47D cells were plated at a density of 5 × 10^4^ cells per well in six-well cell culture plates. After 24 h, cells were treated with dasatinib (1 µM). Dasatinib was purchased from Selleckchem. The preparation and storage of the stock solution and its subsequent use followed the manufacturer’s recommendations. The medium with freshly added dasatinib was replaced every day. Cells were harvested 48 and 72 h after dasatinib treatment and incubated for 30 min–1 h on ice in propidium-iodide (PI) staining solution. Cells were analyzed by a flow cytometer (FACS Canto II, BD Bioscience).

### 4.9. Statistical Analysis

Differences between experimental groups were assessed using Student’s *t*-test (Statistical Analysis System, Release 9.3, SAS Software, Heidelberg, Germany). *p* Values < 0.05 were considered significant.

### 4.10. Antibodies, shRNAs and Primer

Antibodies to detect FOXM1 (#5438), YAP (#14074), LATS1 (#9153), LATS2 (#5888) were purchased from Cell Signaling Technology (Heidelberg, Germany). Antibodies for detection of humen ER alpha (D-12): sc-8005, β-actin (C-4): sc-47778 and p21 (C-19): sc-397 were purchased from Santa Cruz Inc (Heidelberg, Germany). Anti-human RASSF1A [3F3] (ab23950) was purchased from Abcam (Cambridge, UK) and anti-human vinculin was purchased from Sigma-Aldrich (Taufkirchen, Germany). The secondary antibodies polyclonal goat anti-mouse immunoglobulins/HRP and polyclonal goat anti-rabbit immunoglobulins/HRP were purchased from Agilant (Santa Clara, CA, USA). shRNAs against human ER alpha, human FOXM1, human YAP1, human LATS1 and human LATS2 were purchased from Sigma-Aldrich (Taufkirchen, Germany). ER alpha shRNA1: TRCN0000003298, ER alpha shRNA2: TRCN0000003300, FOXM1 shRNA1: TRCN0000015544, FOXM1 shRNA2: TRCN000005546, YAP1 shRNA1: TRCN0000300282, YAP1 shRNA2: TRCN0000107266, LATS1 shRNA: TRCN0000001776, LATS2-1 shRNA: TRCN0000000884, LATS2-2 shRNA: TRCN0000000880. Primer: ER alpha forward: attggccagtaccaatgacaaggg, ER alpha reverse: tatcaatggtgcactggttggtgg, FOXM1 forward: acctgcagctagggatgtgaatct, FOXM1 reverse: aagccactggatgttggataggct, YAP1 forward: tag-ccctgcgtagccagtta, YAP1 reverse: tcatgcttagtccactgtctgt, LATS1 forward: tggtcatattaaattgactgac, LATS1 reverse: ccacatcgacagcttgaggg, LATS2 forward: tagagcagagggcgcggaag, LATS2 reverse: ccaacactccaccagtcacaga, RibPO forward: agacaatgtgggctccaagcagat, RibPO reverse: gcatcatggtgttcttgcccatca. For more details see Supplementary Table S1. 

## 5. Conclusions

Here we show that RASSF1A acts as a tumor suppressor in ERα-expressing breast cancer cells by counteracting ERα expression and function, inhibiting cell cycle progression and inducing senescence. We identified the RASSF1A-mediated modulation of YAP1 as the main cause of suppression of ERα expression after re-introduction of RASSF1A. This notion was confirmed through YAP1 knockdown experiments, which phenocopied the effect of RASSF1A on ERα expression. The knockdown of either LATS1 or LATS2 circumvents the RASSF1A-mediated modulation of YAP1, as well as the inhibition of ERα and the induction of senescence. We therefore conclude that RASSF1A executes its tumor-suppressor functions in ERα-driven breast cancer cells through LATS1 and LATS2, and that mutual interaction between RASSF1A and LATS1 and LATS2 are important for the suppression of ERα+ breast cancer.

## Data Availability

The data presented in this study are available on request from the corresponding author.

## Supplementary Materials

The following are available online at https://www.mdpi.com/article/10.3390/cells10112868/s1, Figure S1: RASSF1A decreases YAP1 protein levels and inhibits FOXM1 and ERα expression. (**A**) Expression of RASSF1A in conditional RASSF1A T47D cells was induced by culturing cells in the presence of 1 μg/mL doxycycline. Cell extracts from induced and non-induced conditional RASSF1A cells were prepared 48 h after doxycycline administration and were analyzed by Western blotting using the indicated antibodies (left panel). Conditional RASSF1A cells were plated at equal densities and grown for 5 days in the presence or absence of doxycycline as indicated. RASSF1A- induced senescence was subsequently monitored by SA-β-gal staining (right panel). Quantification of senescent cells was achieved by counting. Mean values ± s.d. of six independent experiments are shown. *p*-values < 0.05 are indicated by asterisks. Equal numbers of conditional RASSF1A cells were plated on six-well plates and grown for 9 days in the presence or absence of doxycycline as indicated. Quantification of colonies was achieved by counting (right panel). (**B**) RASSF1A downregulates transcription of ERα and FOXM1 but does not change transcription of YAP1. Conditional T47D cells were grown in the presence or absence of 1 μg/mL doxycycline as indicated. mRNA was harvested 48 h after doxycycline administration. ERα, FOXM1 and YAP1 transcript levels were analyzed by quantitative PCR. Mean values ± s.d. of four independent experiments are shown. *p*-values < 0.05 are indicated by asterisks. Figure S2: Pharmacological inhibition of YAP1 leads to induction of cell cycle arrest. MCF7 [(**A**,**C**)] or T47D [(**B**,**D**)] cells were plated on six-well cell culture plates at a seeding density of 5 × 104 per well. MCF7 and T47D cells were cultured in the absence or presence of the indicated concentration of dasatinib. Medium with freshly added dasatinib was replaced every day. Cells were harvested after 48 h of treatment (**A**,**B**) or after 72 h of dasatinib treatment (**C**,**D**). Afterwards cells were used for PI staining and cell cycle analysis. Figure S3: LATS2 inhibition by shRNAs elicits different outcomes depending on the degree of knockdown. (**A**) Stable knockdown of LATS2 was performed in RASSF1A-conditional MCF7 cells using shRNAs (LATS1 and LATS2 kd2) and mRNA was prepared 48 h after lentiviral transduction. Transcript levels of the LATS2 gene was analysed by quantitative PCR. Mean values ± s.d. of three independent experiments are presented. (**B**,**C**) Equal numbers of conditional RASSF1A cells were plated on six-well plates and transduced with equal amounts of lentiviral particle (a) non-targeted shRNA (scramble), (b) shRNA LATS2 kd1 or (c) shRNA LATS2 kd2 lentiviral particles. Quantification of senescent cells was achieved by counting. Mean values ± s.d. of three independent experiments are shown. shRNA LATS2 kd2 was used for the LATS2 knockdown and LATS1+LATS2 double knockdown experiments shown in Figure 4 and Figure 5. Table S1: Additional Materials and Methods information.
