# Supplementary material for: RASSF1A-Mediated Suppression of Estrogen Receptor Alpha (ERα)-Driven Breast Cancer Cell Growth Depends on the Hippo-Kinases LATS1 and 2
Source: Cells. 2021 Oct 24;10(11):2868. doi: 10.3390/cells10112868 (PMC8616147; doi:10.3390/cells10112868)
Supplement: Supplementary file 1 [file cells-10-02868-s001.zip › cells-1285526-Supplementary Figures.pdf]

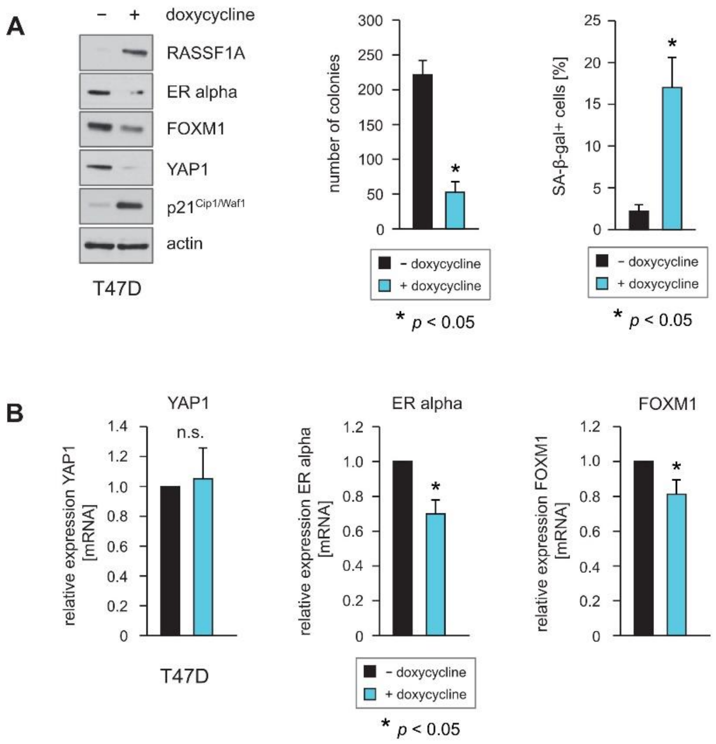

Supplementary Figure S1. RASSF1A decreases YAP1 protein levels and inhibits ERα and FOXM1 expression (A) Expression of RASSF1A in conditional RASSF1A T47D cells was induced by culturing cells in the presence of 1 μg/ml doxycycline. Cell extracts from induced and non-induced conditional RASSF1A cells were prepared 48 h after doxycycline administration and were analyzed by Western blotting using the indicated antibodies (left panel). Conditional RASSF1A cells were plated at equal densities and grown for 5 days in the presence or absence of doxycycline as indicated. RASSF1A-induced senescence was subsequently monitored by SA-β-gal staining (right panel). Quantification of senescent cells was achieved by counting. Mean values ± s.d. of six independent experiments are shown. *P*-values <0.05 are indicated by asterisks. Equal numbers of conditional RASSF1A cells were plated on six-well plates and grown for 9 days in the presence or absence of doxycycline as indicated. Quantification of colonies was achieved by counting (right panel). **(B)** RASSF1A downregulates transcription of ERα and FOXM1 but does not change transcription of YAP1. Conditional T47D cells were grown in the presence or absence of 1 μg/ml doxycycline as indicated. mRNA was harvested 48 h after doxycycline administration. ERα, FOXM1 and YAP1 transcript levels were analyzed by quantitative PCR. Mean values ± s.d. of four independent experiments are shown. *P*-values <0.05 are indicated by asterisks.

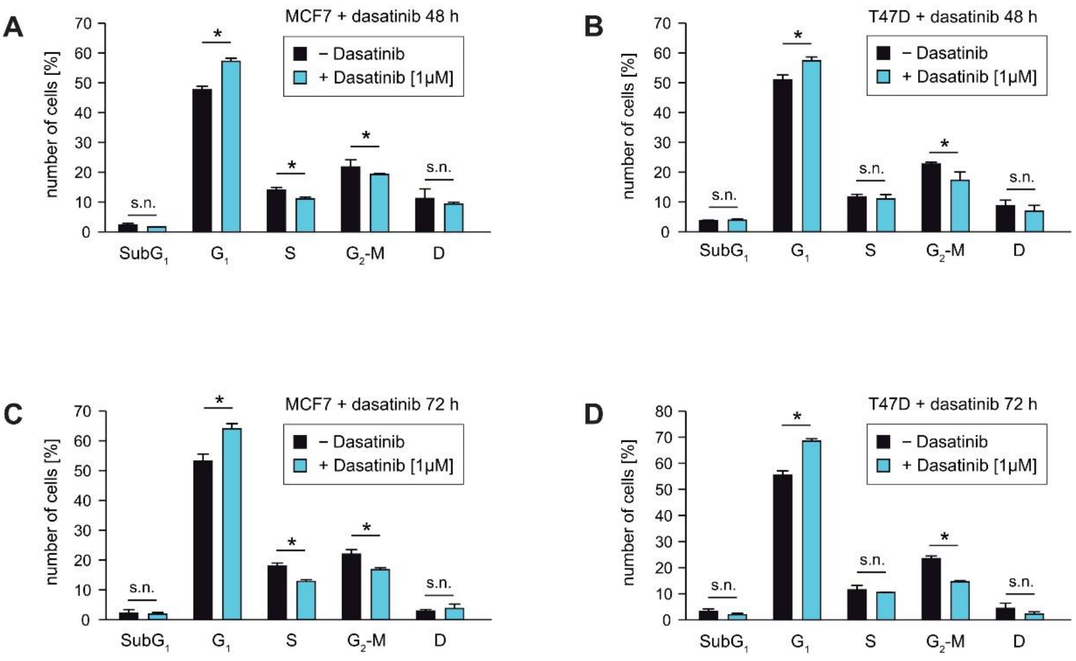

Supplementary Figure S2. Pharmacological inhibition of YAP1 leads to induction of cell cycle arrest. MCF7 **[(A)** and **(C)]** or T47D **[(B)** and **(D)]** cells were plated on six-well cell culture plates at a seeding density of 5 x 10^4 per well. MCF7 and T47D cells were cultured in the absence or presence of the indicated concentration of dasatinib. Medium with freshly added dasatinib was replaced every day. Cells were harvested after 48 h of treatment **(A)** and **(B)** or after 72 h of dasatinib treatment **(C)** and **(D)**. Afterwards cells were used for PI staining and cell cycle analysis.

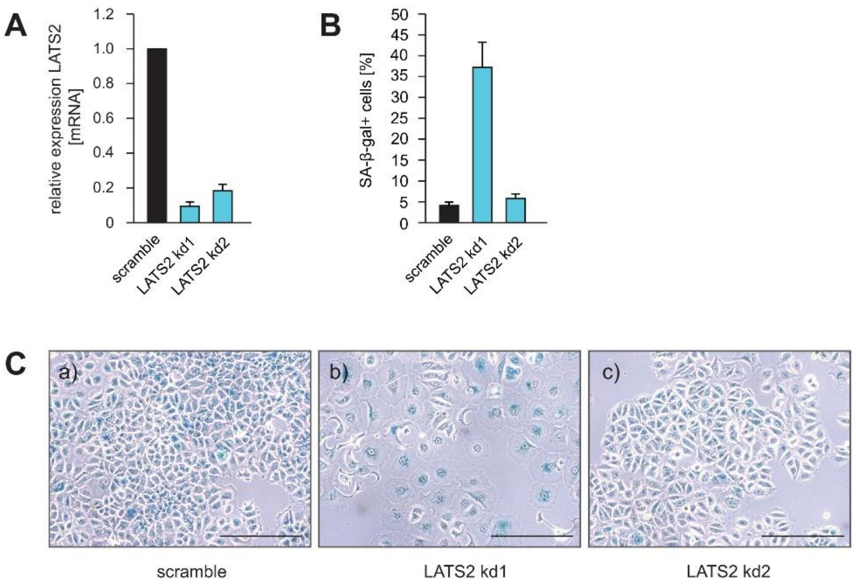

Supplementary Figure S3. LATS2 inhibition by shRNAs elicits different outcomes depending on the degree of knockdown. (A) Stable knockdown of LATS2 was performed in RASSF1A-conditional MCF7 cells using shRNAs (LATS2 kd1 and LATS2 kd2) and mRNA was prepared 48 h after lentiviral transduction. Transcript levels of the LATS2 gene was analyzed by quantitative PCR. Mean values ± s.d. of three independent experiments are presented. **(B)** and **(C)** Equal numbers of conditional RASSF1A cells were plated on six-well plates and transduced with equal amounts of lentiviral particles carrying **(a)** non-targeted shRNA (scramble), **(b)** shRNA LATS2 kd1 or **(c)** shRNA LATS2 kd2 lentiviral particles. For quantification of SA-β-gal positive cells, cells were fixed and stained after 5 days. Bars=100 μm. Quantification of senescent cells was achieved by counting. Mean values ± s.d. of three independent experiments are shown. shRNA LATS2 kd2 was used for the LATS2 knockdown and LATS1+LATS2 double knockdown experiments shown in Figure 4 and 5.
