# Supplementary material for: RASSF1A-Mediated Suppression of Estrogen Receptor Alpha (ERα)-Driven Breast Cancer Cell Growth Depends on the Hippo-Kinases LATS1 and 2
Source: Cells. 2021 Oct 24;10(11):2868. doi: 10.3390/cells10112868 (PMC8616147; doi:10.3390/cells10112868)
Supplement: Supplementary file 1 [file cells-10-02868-s001.zip › cells-1285526-Table S1 Additional Materials and Methods information.pdf]

TableS1. Additional Materials and Methods information.

Antibodies

Primary antibodies

RASSF1A Abcam [3F3] (ab23950), dilution: 1:1000

FOXM1 Cell Signaling # 5438, dilution: 1:500

Estrogen receptor alpha Santa Cruz (D-12) sc-8005, dilution: 1:200

β-actin Santa Cruz (C-4) sc-47778, dilution: 1:200

YAP Cell Signaling # 14074, dilution: 1:1000

LATS1 Cell Signaling # 9153, dilution: 1:1000

LATS2 Cell Signaling # 5888, dilution: 1:1000

p21 Santa Cruz sc-397, dilution: 1:200

vinculin Sigma Aldrich V9131, dilution: 1:1000

Secondary antibodies

Polyclonal Goat Anti-Mouse Immunoglobulins/HRP (Agilant Technologies), dilution: 1:1000

Polyclonal Goat Anti-Rabbit Immunoglobulins/HRP (Agilant Technologies), dilution: 1:2000

shRNAs

shFOXM1 ko-1 pLKO.1 puro Sigma Aldrich TRCN0000015544
5'-ccgggcccaacaggagtctaatcaactcgagttgattagactcctgttgggcttttt-3'

shFOXM1 ko-2 pLKO.1 puro Sigma Aldrich TRCN0000015546
5'-ccgggccaatcgttctctgacagaactcgagttctgtcagagaacgattggcttttt-3'

shYAP1 ko-1 pLKO.1 puro Sigma Aldrich TRCN0000300282
5‘-ccggcccagttaaatgttcaccaatctcgagattggtgaacatttaactgggtttttg-3‘

shYAP1 ko-2 pLKO.1 puro Sigma Aldrich TRCN0000107266
5‘-ccgggccaccaagctagataaagaactcgagttctttatctagcttggtggctttttg-3‘

shLATS1 ko-1 pLKO.1 puro Sigma Aldrich TRCN0000001776
5‘-ccgggaagataaagacactaggaatctcgagattcctagtgtctttatcttcttttt-3‘

shLATS2 ko-1 pLKO.1 puro Sigma Aldrich TRCN0000000884
5‘-ccggctactcgccatacgcctttaactcgagttaaaggcgtatggcgagtagttttt-3‘
(not used for functional analysis, this shRNA induced senescence in approximately 70-80% of kd cells, see **Supplementary Figure 3**)

shLATS2 ko-2 pLKO.1 puro Sigma Aldrich TRCN0000000880
5‘-ccggccgtcgattacttcacttgaactcgagttcaagtgaagtaatcgacggttttt-3‘
(used to investigate whether LATS2 ko can compensate RASSF1A-mediated suppression of YAP1)

Primer

Estrogen Receptor alpha fw	att ggc cag tac caa tga caa ggg
Estrogen Receptor alpha rev	tat caa tgg tgc act ggt tgg tgg
FOXM1 fw	acc tgc agc tag gga tgt gaa tct
FOXM1 rev	aag cca ctg gat gtt gga tag gct
YAP1 fw	tag ccc tgc gta gcc agt ta
YAP1 rev	tca tgc tta gtc cac tgt ctg t
LATS1 fw	tgg tca tat taa att gac tga c
LATS1 rev	cca cat cga cag ctt gag gg
LATS2 fw	tag agc aga ggg cgc gga ag
LATS2 rev	cca aca ctc cac cag tca cag a
RibPO fw	aga caa tgt ggg ctc caa gca gat
RibPO rev	gca tca tgg tgt tct tgc cca tca

Plasmide

p2xFLAGhYAP1-S127A	addgene.org/plasmid #17790 [48].
p2xFLAGhYAP1	addgene.org/plasmid #17791 [48].
